# Predicting 28-day mortality for patients with acute anterior circulation large vessel occlusion stroke following endovascular treatment in neurology intensive care units

**DOI:** 10.3389/fneur.2025.1630773

**Published:** 2025-08-20

**Authors:** Wenjing Qin, Chengcheng Xiao, Jing Yang, Mei Hu, Liying Chang, Yanhan Zhu

**Affiliations:** ^1^Department of Neurology, Xiangyang Central Hospital, Affiliated Hospital of Hubei University of Arts and Science, Xiangyang, China; ^2^Department of Rehabilitation Medicine, Xiangyang Central Hospital, Affiliated Hospital of Hubei University of Arts and Science, Xiangyang, China

**Keywords:** APACHE II, large vessel occlusion stroke, endovascular treatment, intensive care unit, mortality

## Abstract

**Background:**

The clinical utility of the National Institutes of Health Stroke Scale, Glasgow Coma Scale, and modified Rankin Scale scores in predicting prognosis is well established. However, whether the Acute Physiology and Chronic Health Evaluation System II (APACHE II) score can predict mortality in patients with large vessel occlusion stroke (LVOS) admitted to the neurology intensive care unit (NICU) following endovascular treatment (EVT) remains unclear. This study aims to evaluate the ability of the APACHE II score to predict mortality in post-EVT LVOS patients admitted to the NICU.

**Methods:**

This retrospective cohort study enrolled 93 consecutive patients (65 males; mean age, 68.0 years) with acute anterior circulation LVOS who underwent EVT. Patients were categorized into survival and death groups based on their 28-day post-EVT survival status. APACHE II scores of the two groups were compared. Receiver operating characteristic (ROC) curve analysis was employed to assess the sensitivity, specificity, and optimal threshold of APACHE II scores in predicting mortality. Model calibration was assessed using the Hosmer-Lemeshow goodness-of-fit test. Multivariable logistic regression was performed to estimate odds ratios (ORs) for mortality prediction.

**Results:**

Of the 93 enrolled patients, 74 (79.6%) survived and 19 (20.4%) died within 28 days. The death group had significantly higher APACHE II scores than the survival group [(21.84 ± 4.10) points vs. (13.05 ± 5.54) points, *p* < 0.001]. ROC analysis revealed excellent discriminative capacity (AUC 0.912, 95% CI 0.850–0.973), with an optimal threshold of 16.5 points (sensitivity 94.7%, specificity 75.7%). The mortality rate was 1.8% for patients with APACHE II scores <16.5 points and 50.0% for those with APACHE II scores ≥16.5 points. The model demonstrated good calibration (*p* = 0.878). Further, multivariable analysis confirmed both APACHE II scores (OR = 1.239, 95% CI 1.029–1.491, *p* = 0. 023) and cerebral hernia (OR = 11.404, 95% CI 1.507–86.314, *p* = 0. 018) as independent predictors.

**Conclusion:**

APACHE II score assessed within 24 h post-EVT provides robust prediction of 28-day mortality in acute anterior circulation LVOS patients admitted to the NICU.

## 1 Introduction

Large vessel occlusion stroke (LVOS) is associated with high mortality and disability, with reported poor functional outcomes in 60–80% of patients at 3 months post-onset. Since the landmark 2015 trials demonstrated the efficacy and safety of endovascular treatment (EVT) for LVOS, multiple prognostic factors, including age, baseline National Institutes of Health Stroke Scale (NIHSS) score, Alberta Stroke Project Early Computed Tomography Score (ASPECTS), reperfusion grade, collateral status, core infarct size prior to EVT, infarct location, 24-h NIHSS score, final infarct volume, and hemorrhagic transformation (PH1 or PH2 type), have been identified (1–4). For LVOS patients admitted to the neurology intensive care unit (NICU) post-EVT, an objective and accurate assessment of prognosis and prediction of mortality is essential to facilitate doctor–patient communication and make informed medical decisions. In clinical practice, neurological scores [NIHSS, Glasgow Coma Scale (GCS), and modified Rankin Scale (mRS) scores] are generally used to evaluate the severity and prognosis (5–7). However, they fail to account for systemic physiological derangements. Previous studies have reported that patient frailty, comorbidities, and post-stroke complications adversely affect prognosis (3, 8, 9). Thus, finding a comprehensive mortality prediction tool for post-EVT LVOS patients in the NICU, is important.

Acute Physiology and Chronic Health Evaluation System II (APACHE II) score is widely used to predict mortality in critically ill patients within 24 h of ICU admission (10, 11). This scoring system has demonstrated reliability in mortality prediction across multiple conditions including acute pancreatitis, sepsis, and trauma (12–14). Previous studies have also validated its application in stroke (15–17). For patients with acute ischemic stroke, or ischemic stroke patients admitted to ICU, APACHE II score has been shown to be a reliable predictor (18–20). However, the utility of APACHE II scoring system in LVOS patients following EVT remains unevaluated. Therefore, this study aimed to investigate the predictive value of the APACHE II score for 28-day mortality in anterior circulation LVOS patients admitted to the NICU post-EVT. These findings provide an objective clinical tool to guide NICU discharge decisions, identify high-risk patients, inform medical decision-making, and establish evidence-based criteria for post-EVT care pathways and cost-effectiveness in LOVS management.

## 2 Methods

### 2.1 Study design

**Figure 1:**
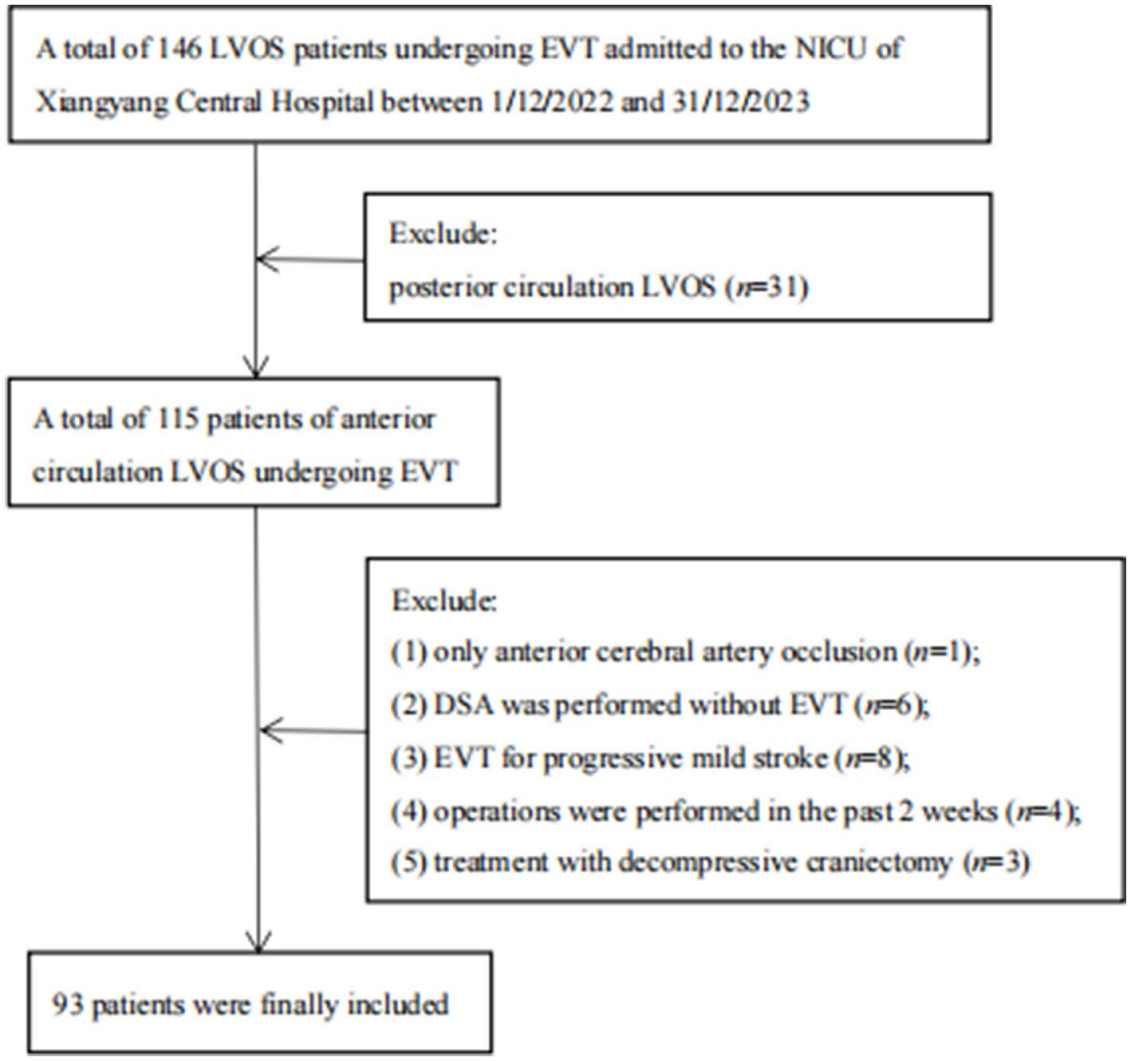
Flowchart of inclusion and exclusion. DSA, digital subtraction angiography; EVT, endovascular treatment; LVOS, large vessel occlusion stroke; NICU, neurology intensive care unit.

We conducted a retrospective study of 93 patients with anterior circulation LVOS from 146 post-EVT patients who were admitted to the NICU, Xiangyang Central Hospital, Affiliated Hospital of Hubei University of Arts and Science between November 2022 and December 2023 (Figure 1). This study was approved by our hospital’s Ethics Committee (2023–074).

### 2.2 Study objects

The inclusion criteria were as follows: (i) age > 18 years; (ii) symptom onset within 24 h; (iii) meeting the indications and contraindications of EVT for acute ischemic stroke (21); (iv) computed tomography angiography (CTA) images showing anterior circulation large vessel occlusion (occlusion in the internal carotid artery and/or middle cerebral artery), which led to stroke; (v) digital subtraction angiography (DSA) showing anterior circulation large vessel occlusion (occlusion in the internal carotid artery and/or middle cerebral artery) pre-EVT; (vi) head computed tomography (CT) scans were performed post-EVT.

The exclusion criteria were as follows: (i) mRS score > 1 point pre-EVT; (ii) posterior circulation large vessel occlusion or isolated anterior cerebral artery occlusion on CTA/DSA; (iii) DSA performed without subsequent EVT; (iv) EVT performed for progressive mild stroke with large vessel occlusion; (v) decompressive craniectomy during the course of the disease; (vi) major surgery performed in the past 2 weeks such as coronary stent operations and surgical operations; (vii) incomplete clinical, laboratory, or imaging data.

Based on the survival status at 28 days post-EVT, the patients were divided into survival and death groups.

### 2.3 Data collection

Clinical data collection involved gathering information on demographics (age and sex), vascular risk factors (hypertension, atrial fibrillation, stroke history, type 2 diabetes mellitus, high serum cholesterol, ischemic heart disease, smoking, and alcohol consumption), baseline blood pressure (both systolic and diastolic), body mass index, and laboratory tests (including serum total cholesterol, low-density lipoprotein cholesterol [LDL-C], triglycerides, fasting blood glucose, D-dimer, homocysteine, glycosylated hemoglobin, white blood cell count, neutrophil-to-lymphocyte ratio [NLR], creatinine, N-terminal pro-B-type natriuretic peptide, and albumin). Additionally, data on the offending vessels (the internal carotid artery or middle cerebral artery) and its side (left or right), baseline NIHSS and 24-h NIHSS scores, baseline GCS and 24-h GCS scores, presence of cerebral hernia, hemorrhagic transformation, APACHE II score, and predicted mortality rate (R-value) were collected.

According to the ECASS III classification, hemorrhagic transformation was assessed using post-EVT head CT imaging and categorized into four types: HI1 was defined as small petechial hemorrhages along the margins of the infarct; HI2 referred to confluent petechial hemorrhages within the infarct zone without space-occupying effect; PH1 was characterized by hematomas involving <30% of the infarct area with mild mass effect; while PH2 represented hematomas occupying >30% of the infarct area with significant mass effect or hemorrhages occurring outside the infarct zone (22).

The APACHE II scoring system comprises three components: age, acute physiology score (APS), and chronic health score (CHS), with a maximum total score of 71 points. The APS incorporates 12 physiological parameters: body temperature, blood pressure (systolic and diastolic), heart rate, respiratory rate, pH value, arterial partial oxygen pressure, serum sodium, potassium, creatinine, hematocrit, white blood cell count, and GCS score. In this study, GCS scores were assessed based on neurological function 24 h post-NICU admission. The best guess principle was applied for sedated patients. For patients with artificial airways, the verbal response score was documented as 5–3–1. When asymmetric limb strength was present, the score was based on the less-affected side. All other APS parameters were evaluated using the most abnormal values recorded within the first 24 h. The CHS assessed pre-existing dysfunction in five organ systems: hepatic, cardiovascular, respiratory, renal, and immunologic. The predicted mortality rate (*R*-value) was calculated from the APACHE II score.

### 2.4 Statistical analysis

Shapiro–Wilk test assessed the normal distribution of quantitative data. Normally distributed data were presented as mean ± standard deviation and compared using independent-samples *t-*test. Non-normally distributed data were presented as median (lower quartile and upper quartile) and compared using Mann–Whitney *U*-test. Count data were presented as percentages and analyzed with Pearson’s Chi-square test. The sensitivity, specificity, and optimal threshold of APACHE II score were assessed using the receiver operating characteristic (ROC) curve and area under the curve (AUC) analysis. The Hosmer-Lemeshow goodness-of-fit test evaluated the calibration of APACHE II mortality predictions. Binary, multivariable logistic regression models identified factors associated with 28-day mortality. All statistical tests were two-sided, with *p* < 0.05 considered statistically significant. The analysis was performed using the SPSS software package (version 26.0; IBM Corp., Armonk, NY, United States).

## 3 Results

Ninety-three anterior circulation LVOS patients admitted to the NICU post-EVT were included. They were 65 males (69.9%) and 28 females (30.1%), with a mean age of 67.96 ± 11.52 years (range: 35–93 years). Seventy-four patients (79.6%) survived at 28-day (survival group), including 27 (36.5%) with internal carotid artery occlusion and 47 (63.5%) with isolated middle cerebral artery occlusion. The remaining 19 patients (20.4%) died (death group), including 6 (31.6%) with internal carotid artery occlusion and 13 (68.4%) with isolated middle cerebral artery occlusion (Table 1).

**Table 1:** Demographic and baseline data of the survival group and the death group.

Variable	Total patients (*n* = 93)	The survival status after 28 days of onset			
		The survival group (*n* = 74)	The death group (*n* = 19)	Statistical value	*P*
Demography					
Age (year, $\bar{x}$ ±*s*)	67.96 ± 11.52	66.70 ± 11.95	72.84 ± 8.24	−2.110	0.038
Male (*n,* %)	65 (69.9)	55 (74.3)	10 (52.6)	3.381	0.066
Vascular risk factors (*n,* %)					
Hypertension	60 (64.5)	46 (62.2)	14 (73.7)	0.877	0.349
Atrial fibrillation	37 (39.8)	25 (33.8)	12 (63.2)	5.445	0.020
Stroke history	25 (26.9)	17 (23.0)	8 (42.1)	2.815	0.093
Type 2 diabetes	14 (15.1)	9 (12.2)	5 (26.3)	1.391	0.238
High serum cholesterol	24 (25.8)	20 (27.0)	4 (21.1)	0.056	0.813
Ischemic heart disease	25 (26.9)	17 (23.0)	8 (42.1)	2.815	0.093
Smoking	42 (45.2)	35 (47.3)	7 (36.8)	0.667	0.414
Alcohol consumption	36 (38.7)	30 (40.5)	6 (31.6)	0.512	0.474
Baseline blood pressure (mmHg, $\bar{x}$ ±*s*)					
Systolic blood pressure	150.90 ± 30.03	150.31 ± 28.03	153.21 ± 37.65	−0.374	0.710
Diastolic blood pressure	87.97 ± 17.66	88.15 ± 16.61	87.26 ± 21.77	0.194	0.847
BMI (Kg/m^2^, $\bar{x}$ ±*s*)	23.93 ± 3.45	24.11 ± 3.29	23.22 ± 4.04	1.005	0.317
Laboratory tests					
Total cholesterol (mmol/L, $\bar{x}$ ±*s*)	4.26 ± 0.96	4.18 ± 0.92	4.57 ± 1.07	−1.610	0.111
LDL-C (mmol/L, $\bar{x}$ ±*s*)	2.38 (1.90 ~ 2.97)	2.38 (1.89 ~ 2.91)	2.68 (1.96 ~ 3.20)	−0.310	0.757
Triglycerides (mmol/L; *M, IQR*)	1.02 (0.75 ~ 1.49)	0.95 (0.73 ~ 1.38)	1.10 (0.81 ~ 1.71)	−1.277	0.202
Fasting blood glucose (mmol/L; *M, IQR*)	7.79 (5.86 ~ 9.34)	7.49 (5.85 ~ 8.90)	8.58 (5.87 ~ 12.63)	−1.753	0.080
D-dimer (mg/L; *M, IQR*)	0.43 (0.18 ~ 1.35)	0.35 (0.17 ~ 1.31)	0.50 (0.28 ~ 1.59)	−1.072	0.284
Hcy (μmol/L; *M, IQR*)	15.20 (12.45 ~ 18.45)	15.30 (12.48 ~ 18.83)	15.20 (12.20 ~ 18.20)	−0.415	0.678
Glycosylated hemoglobin(%; *M*, *IQR*)	5.70 (5.30 ~ 6.10)	5.70 (5.30 ~ 6.03)	5.90 (5.20 ~ 6.40)	−0.806	0.420
White blood cell (*10^9^/L, $\bar{x}$ ±*s*)	8.81 (6.84 ~ 11.62)	8.83 (6.86 ~ 11.40)	8.81 (5.63 ~ 12.49)	−0.386	0.700
NLR (%; *M, IQR*)	5.48 (2.26 ~ 8.70)	5.49 (2.28 ~ 8.16)	4.09 (1.96 ~ 9.19)	−0.029	0.977
Creatinine (μmol/L, $\bar{x}$ ±*s*)	73.60 (61.45 ~ 88.80)	72.45 (61.08 ~ 87.35)	77.00 (61.70 ~ 97.50)	−0.600	0.548
N-terminal pro-B-type natriuretic peptide (pg/mL; *M, IQR*)	827.60 (160.95 ~ 1715.00)	534.45 (140.98 ~ 1486.50)	1512.00 (706.70 ~ 3516.00)	−2.315	0.021
Albumin (g/L; *M, IQR*)	37.10 (34.85 ~ 39.30)	37.15 (34.98 ~ 39.13)	37.00 (34.50 ~ 40.90)	−0.543	0.587
Baseline NIHSS (point, $\bar{x}$ ±*s*)	19.00 (16.00 ~ 21.00)	18.00 (16.00 ~ 21.00)	19.00 (17.00 ~ 28.00)	−1.739	0.082
24-h NIHSS (point; *M, IQR*)	14.00 (9.00 ~ 22.00)	12.00 (8.75 ~ 17.25)	33.00 (30.00 ~ 34.00)	−6.304	<0.001
Baseline GCS (point, $\bar{x}$ ±*s*)	9.00 (8.00 ~ 11.00)	9.50 (8.00 ~ 11.00)	8.00 (6.00 ~ 9.00)	−2.370	0.018
24-h GCS (point; *M, IQR*)	11.00 (7.00 ~ 14.00)	12.50 (10.00 ~ 14.00)	5.00 (3.00 ~ 6.00)	−6.010	<0.001
Responsible blood vessel (*n,* %)				0.159	0.690
Internal carotid artery	33 (35.5)	27 (36.5)	6 (31.6)		
Middle cerebral artery	60 (64.5)	47 (63.5)	13 (68.4)		
Side of responsible vessel (*n,* %)				0.851	0.356
Left	55 (59.1)	42 (56.8)	13 (68.4)		
Right	38 (40.9)	32 (43.2)	6 (31.6)		
Cerebral hernia (*n*, %)	13 (14.0)	2 (2.7)	11 (57.9)	33.847	<0.001
Hemorrhagic transformation (*n*, %)	39 (41.9)	25 (33.8)	14 (73.7)	9.885	0.002
HI1	8 (8.6)	7 (9.5)	1 (5.3)	0.015	0.902
HI2	9 (9.7)	9 (12.2)	0 (0)	1.356	0.244
PH1	6 (6.5)	4 (5.4)	2 (10.5)	0.082	0.774
PH2	16 (17.2)	5 (6.8)	11 (57.9)	24.281	<0.001
APACHE II score (point, $\bar{x}$ ±*s*)	14.85 ± 6.35	13.05 ± 5.54	21.84 ± 4.10	−6.467	<0.001
*R*-value (%; *M, IQR*)	11.71 (6.89 ~ 19.56)	10.28 (6.89 ~ 15.57)	29.90 (21.59 ~ 39.80)	−5.585	<0.001

APACHE II, acute physiology and chronic health evaluation system II; BMI, body mass index; GCS, Glasgow coma scale; HI, hemorrhagic infarction; Hcy, homocysteine; IQR, interquartile range; LDL-C, low density lipoprotein cholesterol; M, median; NLR, neutrophil/lymphocyte ratio; NIHSS, National Institutes of Health Stroke Scale; PH, parenchymal hemorrhage.

The two groups showed no significant differences in terms of sex distribution, specific vascular risk factors (hypertension, stroke history, type 2 diabetes mellitus, high serum cholesterol, ischemic heart disease, smoking, and alcohol consumption), baseline blood pressure, body mass index, certain laboratory tests (total cholesterol, LDL-C, triglycerides, fasting blood glucose, D-dimer, homocysteine, glycosylated hemoglobin, white blood cell count, NLR, creatinine, and albumin), the offending vessel (the internal carotid artery or isolated middle cerebral artery) and its side (left or right), as well as baseline NIHSS score (all *p* > 0.05).

Patients in the death group were older than those in the survival group (*p* = 0.038) and had a higher rate of atrial fibrillation (*p* = 0.020). The death group also demonstrated elevated N-terminal pro-B-type natriuretic peptide levels (*p* = 0.021) and more severe neurological impairment, as evidenced by higher 24-h NIHSS scores (*p* < 0.001) and lower GCS scores both at baseline (*p* = 0.018) and 24-h (*p* < 0.001). Notably, cerebral hernia as a life-threatening complication occurred more frequently in death group (*p* < 0.001). And hemorrhagic transformation was more common in the death group (*p* = 0.002), with PH2 type hemorrhagic transformation being particularly prevalent (*p* < 0.001) (Table 1).

APACHE II scores were significantly higher in the death group (21.84 ± 4.10 points) compared to the survival group (13.05 ± 5.54 points; *p* < 0.001). Similarly, the R-values were higher in the death group [29.90% (21.59, 39.80%)] than the survival group [10.28% (6.89, 15.57%); *p* < 0.001] (Table 1).

**Figure 2:**
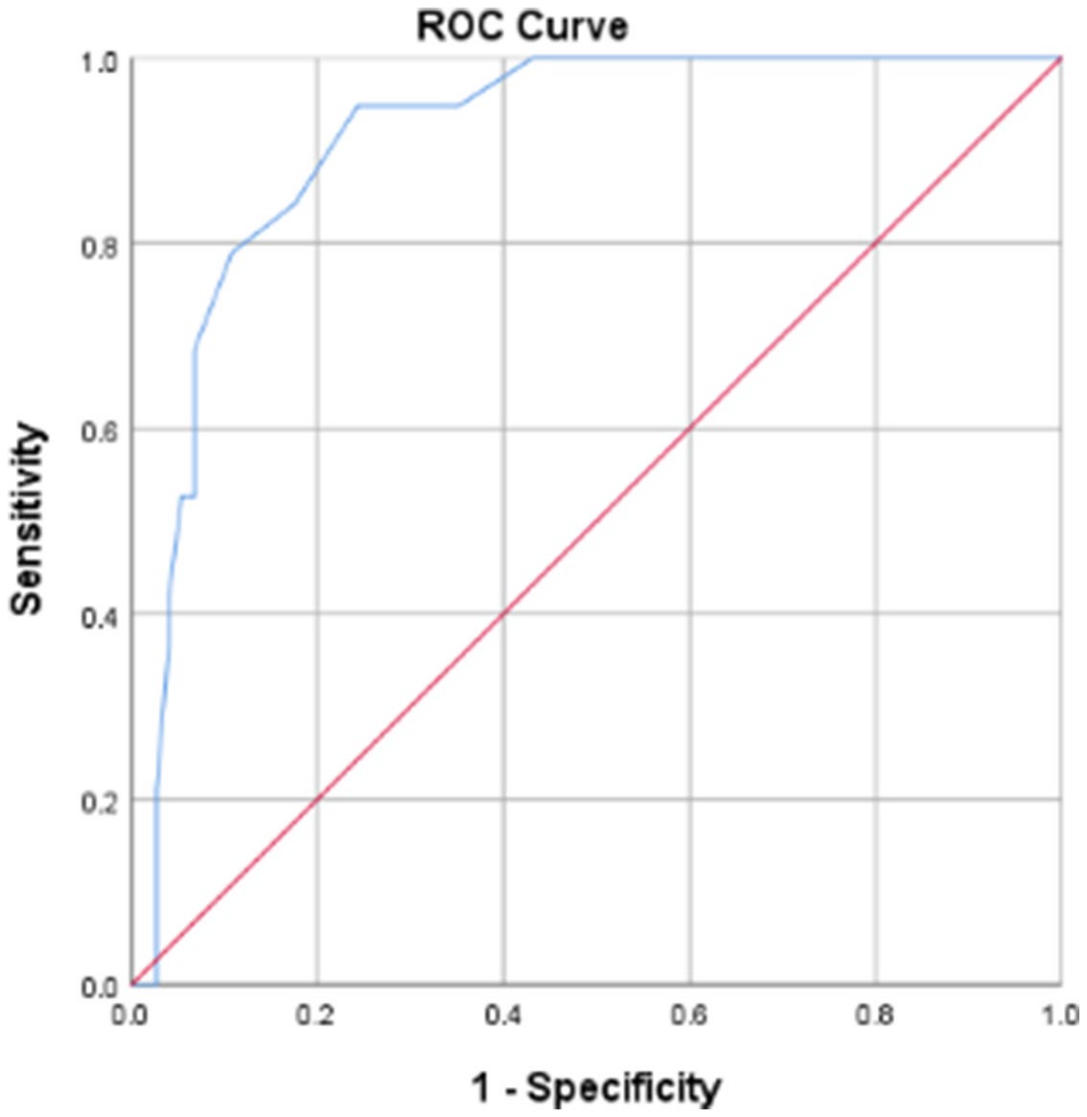
ROC curve of predictive mortality for APACHE II score. APACHE II, acute physiology and chronic health evaluation system II; ROC curve, receiver operating characteristic curve.

The AUC value was 0.912 (95% CI 0.850–0.973), suggesting the excellent discriminative ability of APACHE II scoring system for mortality prediction in anterior circulation LVOS patients post-EVT (Figure 2). ROC analysis identified an optimal threshold of 16.5 points (Youden’s index), achieving 94.7% sensitivity and 75.7% specificity. Patients with scores <16.5 points were classified into the low-risk group, with a fatality rate of 1.8%. Conversely, patients with scores ≥16.5 points were placed in the high-risk group, with a fatality rate of 50.0%.

The Hosmer-Lemeshow goodness-of-fit test demonstrated excellent agreement between predicted and observed mortality (*p* = 0.878), indicating strong calibration of APACHE II scores (Table 2).

**Table 2:** Contingency table of Lemeshow-Hosmer goodness-of-fit test for APACHE II scores.

Step 1	Group				
	The survival group		The death group		Total
	Observed	Expected	Observed	Expected	
1	9	8.934	0	0.066	9
2	6	5.872	0	0.128	6
3	10	9.692	0	0.308	10
4	10	9.555	0	0.445	10
5	7	6.508	0	0.492	7
6	6	6.359	1	0.641	7
7	8	6.983	0	1.017	8
8	5	5.645	2	1.355	7
9	8	8.047	3	2.953	11
10	5	6.405	13	11.595	18

APACHE II, acute physiology and chronic health evaluation system II.

Multivariable logistic regression analyses were performed to identify independent predictors of 28-day mortality, with survival status as the dependent variable and variables with *p* < 0.05 in Table 1 as independent variables. We constructed three models. Model 1 included all significant risk factors, showing 24-h NIHSS as an independent risk factor (OR = 1.726, 95% CI 1.154–2.581, *p* = 0. 008). In Model 2, we excluded two collinear variables, 24-h NIHSS (variance inflation factor (VIF) = 8.847) and 24-h GCS (VIF = 8.409). It revealed that APAHCE II scores (OR = 1.239, 95% CI 1.029–1.491, *p* = 0. 023) and cerebral hernia (OR = 11.404, 95% CI 1.507–86.314, *p* = 0. 018) were significant risk factors. In Model 3, we converted the APAHCE II scores from a continuous variable in model 2 into a categorical variable with the threshold value of 16.5 points. APAHCE II scores (OR = 20.460, 95% CI 2.000–209.263, *p* = 0. 011) and cerebral hernia (OR = 12.406, 95% CI 1.847–83.338, *p* = 0. 010) were independent risk factors in LVOS patients after EVT (Table 3).

**Table 3:** The 28-day mortality odds ratios for LVOS following EVT.

Variable	Odds ratios for 28-day mortality		
	Odds ratio	95% CI	*P*
Model 1			
24-h NIHSS	1.726	1.154–2.581	0.008
Model 2			
APAHCE II score	1.239	1.029–1.491	0.023
Cerebral hernia	11.404	1.507–86.314	0.018
Model 3			0.020
APAHCE II score	20.460	2.000–209.263	0.011
Cerebral hernia	12.406	1.847–83.338	0.010

LVOS, large vessel occlusion stroke; EVT, endovascular treatment; CI, confidence interval; NIHSS, National Institutes of Health Stroke Scale; APACHE II, acute physiology and chronic health evaluation system II.

## 4 Discussion

In 1985, Knaus et al. first proposed the APACHE II scoring system (10). The system is simple to operate and can assess the mortality of each individual patient. Subsequently, APACHE II has become the most commonly used and authoritative scoring system in ICUs. With the development of NICU, the application of APACHE II in patients with critical neurological diseases has gradually increased (20). Moon et al. reported a significant correlation between APACHE II scores and mortality in ICU-admitted acute stroke patients (16). Rordorf et al. analyzed 63 ischemic stroke patients who were admitted to ICU and identified that APACHE II scores were a key predictor of death (19). In the present study, we found that APACHE II scores were a strong predictor of 28-day mortality among anterior circulation LVOS patients admitted to the NICU after EVT, with high discrimination and good calibration. Moreover, each 1-point increase in APACHE II score elevated 28-day mortality odds by 23.9%. These results address the research gap of ascertaining the use of APACHE II scores to predict post-EVT mortality in LVOS patients managed in NICU.

Currently, there is no unified critical APACHE II score for predicting mortality in ischemic stroke. In our study, the optimum threshold was 16.5 points, with a sensitivity of 91.7% and specificity of 86.2%. Patients with APACHE II scores <16.5 had a 28-day mortality rate of 1.8%, whereas those scoring ≥16.5 had a rate of 50.0%. These results partially align with Rordorf et al.’s findings, which proposed a cutoff of 18 points (19). Similar to their study, no deaths occurred in patient with scores <9 points. However, their reported mortality rate for scores ≥9 (43%) was higher than our observed rate (23.2%). This discrepancy may be attributed to differences in patient demographics or treatment protocols. Notably, in this study, patients with APACHE II scores ≥16.5 had a 19.46-fold increased risk of 28-day mortality post-EVT compared to patients with scores <16.5. This cutoff effectively stratified high-risk patients. Importantly, concurrent cerebral hernia in patient with scores ≥16.5 further escalated mortality risk.

Our study demonstrated that APACHE II scores effectively predicted 28-day mortality in NICU-admitted anterior circulation LVOS patients post-EVT. We recognize the following advantages: (i) The APS incorporates GCS, a validated predictor of post-EVT survival in ischemic stroke (23). Consistent with this, our death group showed significantly lower GCS scores at baseline and 24-h. (ii) In addition to GCS, the APS includes 11 physiological parameters. Among these, abnormal blood pressure, leukocytosis, and fever caused by infection, have been reported to be associated with poor clinical outcomes in ischemic stroke patient (24–26). (iii) The APACHE II scoring system comprises age and pre-existing heart failure, which have been shown to predict post-EVT mortality (27, 28). In the present study, patients in the death group were significantly older, had a higher prevalence of atrial fibrillation, and elevated N-terminal pro-B-type natriuretic peptide levels.

However, the APACHE II has several limitations: (i) No indicative parameter for evaluating the severity of neurological impairment such as NIHSS, an international well-validated scale, is available. This is particularly critical as both previous studies and our current data consistently demonstrate significantly higher 24-h NIHSS scores in the death group (5). We therefore propose combining APACHE II with 24-h NIHSS to improve prediction accuracy. (ii) The score fails to account for lesion location-specific prognostic differences (29). (iii) APACHE II captures the worst values within the first 24 h of ICU admission, potentially overlooking dynamic prognostic changes. However, recent evidence has suggested serial assessments at multiple time points may improve predictive accuracy (30).

## 5 Limitations

The study lacked data on stroke etiology, pre-EVT intravenous thrombolysis administration, door-to-puncture time, puncture to recanalization time, and the EVT procedural details. These variables have impacts on mortality. And, our reported mortality rates may have been affected by early care withdrawal decisions, including hospice transfer or mechanical ventilation discontinuation within 28 days of NICU admission. These limitations underscore the need for future multicenter, prospective studies with longer follow-up periods.

## 6 Conclusion

Anterior circulation LVOS is associated with a high 28-day mortality rate of 20.4% following EVT. Age, history of atrial fibrillation, level of N-terminal pro-B-type natriuretic peptide, 24-h NIHSS, baseline GCS and 24-h GCS, cerebral hernia complication, and hemorrhagic transformation are factors influencing mortality. The APACHE II scoring system effectively predicts 28-day mortality, with an optimal cutoff score of 16.5 points. We recommend combining the APACHE II score with the 24-h NIHSS and cerebral hernia for improved prognostic stratification. These findings will be helpful for the clinical evaluation of stroke patients, identification of patients who are at high risk of death, and improvement of doctor-patient communication and medical decision-making, especially in clinical settings.

## Data Availability

The original contributions presented in the study are included in the article/supplementary material, further inquiries can be directed to the corresponding author/s.
